# VEXAS syndrome as a mimicker of ANCA-associated vasculitis

**DOI:** 10.1093/rap/rkae116

**Published:** 2024-09-25

**Authors:** Franco Murillo-Chavez, Brendan Antiochos

**Affiliations:** Department of Medicine, Sinai Hospital of Baltimore, Baltimore, MD, USA; Division of Rheumatology, Department of Medicine, Johns Hopkins University School of Medicine, Baltimore, MD, USA

**Keywords:** VEXAS, ANCA, AAV, vasculitis

## Abstract

**Objectives:**

Differentiating VEXAS syndrome from cases of canonical forms of primary vasculitis remains a significant clinical challenge, particularly for ANCA-associated vasculitis (AAV). We reviewed the clinical features of VEXAS as an AAV mimicker, while adding three new cases to the existing literature.

**Methods:**

We identified three cases of VEXAS with an AAV phenotype in our institution. We performed a comprehensive literature search of available similar cases and summarized and compared the findings. Inclusion criterion was a positive *UBA1* mutation analysis.

**Results:**

Patient 1 was referred for evaluation of eosinophilic granulomatosis with polyangiitis (GPA), but had no active respiratory symptoms, despite CT imaging showing widespread ground-glass opacities. Patient 2 had no history of sinus disease, despite being referred under the diagnostic construct of limited GPA. Patient 3 developed a novel inflammatory syndrome suspected to represent GPA. Six other cases were identified upon literature review. In all the cases, the most common findings were pulmonary infiltrates (67%), skin involvement (55%) and ocular manifestations (44%). Additionally, 44% of cases had renal involvement, with half of them displaying kidney lesions resembling the typical AAV pattern.

**Conclusion:**

VEXAS can mimic different phenotypes of AAV and should be considered in atypical AAV presentations, especially when refractory to multiple treatments. Further studies are needed to explore the immunologic basis for an AAV phenotype within the spectrum of VEXAS.

Key messages

- VEXAS can present as an atypical and/or refractory ANCA-associated vasculitis phenotype.
- Rheumatologists should consider the possibility of VEXAS in cases of AAV with atypical kidney biopsy.

## Introduction

The vacuoles, E1 enzyme, X-linked, autoinflammatory, somatic (VEXAS) syndrome was first described in a cohort of 25 men with a treatment-refractory inflammatory syndrome who possessed a previously unidentified somatic mutation affecting methionine-41 (p.Met41) in the *UBA1* gene, resulting in three probable amino acid substitutions (p.Met41Val, p.Met41Leu or p.Met41Thr) [1]. This mutation is linked to dysregulation of innate immune signalling in the affected myeloid cell compartment [2], resulting in a wide array of inflammatory and haematological clinical features in numerous organ systems [3]. Since its initial description, there have been increasing reports of patients who were initially diagnosed with a rheumatologic disease and later were diagnosed with VEXAS.

It has been postulated that rheumatologists should consider a diagnosis of VEXAS syndrome when faced with cases of older men (after their fifth decade of life) with a refractory adult-onset autoinflammatory syndrome with multiorgan involvement, typically including macrocytosis, recurrent fever, non-vasculitic skin lesions (neutrophilic dermatosis, livedo reticularis, inflammatory nodules, panniculitis and ulcerations, among others), asymmetric mono-/oligoarthritis, relapsing polychondritis, pulmonary involvement and any type of vasculitis [4]. The most commonly reported vasculitis in VEXAS is leukocytoclastic vasculitis; however, there have been reports of other forms of small vessel vasculitis, including IgA vasculitis and ANCA-associated vasculitis (AAV) [5]. Differentiating VEXAS from cases of canonical forms of primary vasculitis remains a significant clinical challenge, particularly for AAV, which can manifest in the same organ systems with nearly identical clinical findings. Here, we review the clinical features of VEXAS as an AAV mimicker, while adding three new cases to the existing literature.

## Methods

We identified three cases of VEXAS syndrome that had a presumptive diagnosis of AAV within our practice. We reviewed the electronic medical records of each case and extracted the clinical data and summarized the findings to compare them with the available published cases.

We performed a comprehensive literature search to identify cases with a diagnosis of VEXAS syndrome and AAV. We searched MEDLINE via PubMed for all articles in English or Spanish from 1 January 2020 to 5 January 2024 using the key words or mesh terms ‘VEXAS’, ‘AAV’, ‘antineutrophil cytoplasmic antibody (ANCA)’, ‘ANCA’, ‘cytoplasmic ANCA (cANCA)’, ‘perinuclear ANCA (pANCA)’, ‘granulomatosis with polyangiitis (GPA)’, ‘microscopic polyangiitis (MPA)’, ‘myeloperoxidase (MPO)’, ‘proteinase 3 (PR3)’ and ‘eosinophilic granulomatosis with polyangiitis (EGPA)’. Only reports that had a *UBA1* mutation analysis were included.

The study was based on a retrospective review of medical records of patients diagnosed with VEXAS syndrome. Informed consent of patients and ethics approval was not necessary, as no active intervention was carried out and patient anonymity was maintained.

## Results

### Patient 1

A 72-year-old male with a remote history of nasal polyps and chronic sinusitis presented to outpatient dermatology for evaluation of recurrent nodular skin lesions in the setting of a recent history of weight loss and fever. Skin biopsy showed medium vessel vasculitis with eosinophils present in the vessel walls. Peripheral blood counts demonstrated a total eosinophil count maximum of 690/mm^3^. Prednisone was initiated at a dose of 40 mg/day, with resolution of both fevers and cutaneous lesions, and the patient was then referred to our centre for consideration of a diagnosis of EGPA. Upon tapering of prednisone to <20 mg/day, both fevers and cutaneous vasculitis returned, along with elevation in acute phase reactants. CT of the chest was obtained, demonstrating upper lobe predominant ground-glass opacities (GGOs) in a bronchovascular distribution. Despite the extensive nature of the GGOs, the patient had no respiratory symptoms of cough, wheezing or dyspnoea, which was felt highly unusual for EGPA. Sinus symptoms were similarly absent and ANCA serologies were negative. Despite the use of azathioprine, methotrexate and mepolizumab, the patient demonstrated persistent dependency on glucocorticoids, leading to an eventual consideration of VEXAS syndrome as an alternate diagnosis. Next-generation sequencing (NGS) performed on peripheral blood revealed a pathogenic *UBA1* mutation (p.Met41Thr), establishing the diagnosis of VEXAS syndrome. Interestingly, he did not demonstrate macrocytosis or other features of myelodysplastic syndrome at the time of diagnosis, but developed macrocytosis 2 years after his initial presentation with cutaneous vasculitis. The patient had an excellent response to tocilizumab, with resolution of skin lesions and pulmonary infiltrates and normalization of inflammatory markers while tapering prednisone to a current dose of 12 mg/day.

### Patient 2

An 88-year-old male presented prior to the initial description of VEXAS syndrome with right eye pain and vision changes associated with periorbital oedema. An MRI of the orbits demonstrated soft tissue swelling in the periorbital and maxillary regions with perineural enhancement of the right optic nerve sheath. Laboratory studies demonstrated the presence of mild macrocytic anaemia and elevated inflammatory markers (ESR 125 mm/h and CRP 14.8 mg/dl). The patient also had a history suggestive of relapsing polychondritis (RP), with features of a seronegative inflammatory arthritis and inflammation of the auricular cartilage. In addition to RP, a diagnosis of GPA was also considered, although ANCA serology testing was negative. Initial treatment was given in the form of methylprednisolone pulses followed by oral steroid taper and methotrexate. On follow-up, he demonstrated resolution in his periorbital swelling and eye pain, but macrocytosis was a persistent finding. Recurrence of arthralgia occurred with attempts to completely withdraw steroids. Subsequent to its reporting, VEXAS syndrome was considered as a unifying diagnosis and a Sanger sequencing–based test demonstrated the presence of a p.Met4Leu *UBA1* mutation in peripheral blood. The patient’s course was characterized by symptomatic response to long-term, low-dose (5 mg) steroids. He later died from complications of a pre-existing heart failure.

### Patient 3

A 77-year-old male with a prior medical history of pemphigus vulgaris (treated with rituximab), presented for evaluation of suspected GPA. He had upper respiratory tract symptoms characterized by intermittent productive cough, chest congestion and hoarseness, which were associated with headaches, fatigue and intermittent fever. He was first prescribed a short course of azithromycin and prednisone with relative improvement, but shortly thereafter presented with left orbital swelling, joint pain and kidney injury. A chest CT revealed multiple small bilateral lung nodules associated with bilateral pleural effusions, while lab studies demonstrated rapidly progressive elevation of serum creatinine. Urinalysis showed no haematuria or leukocyturia, with just trace proteinuria on dipstick. c-ANCA was positive at a titre of 1:40, while ELISA was negative for anti-PR3 and anti-MPO. Inflammatory markers were elevated (ESR 100 mm/h and CRP 8 mg/dl). A presumptive diagnosis of GPA was made and he was initially started on pulse dose steroids. However, a kidney biopsy was obtained and did not show findings compatible with GPA, instead demonstrating glomerulosclerosis, tubular injury, tubulointerstitial scarring, moderate arteriolosclerosis and hyalinosis with negative IF and no evidence of vasculitis. Given his atypical course, negative PR3 ELISA and lack of evidence of GPA on renal biopsy, an alternate diagnosis of VEXAS syndrome was considered; NGS from peripheral blood was positive for a p.Met41Thr mutation in *UBA1*. Unfortunately, the patient contracted *Legionella* pneumonia while being treated with glucocorticoids and developed respiratory failure leading to his death.

## Discussion

VEXAS syndrome can manifest as multiple autoimmune and autoinflammatory disorders, including AAV and other forms of vasculitis [5, 6]. Because the vasculitides are themselves relatively uncommon, the intersection between VEXAS and vasculitis creates a uniquely challenging diagnostic scenario. In our study, we identified six previously described cases of AAV occurring in the context of VEXAS syndrome and contributed three more from our centre to the existing literature (Table 1). Interestingly, GPA emerged as the most frequently reported AAV phenotype, with seven cases in total (Table 1). Importantly, all three cases from our centre exhibited clinical features that were atypical for AAV. Patient 1 was referred for evaluation of EGPA but had no active respiratory symptoms despite CT imaging showing widespread GGOs. Patient 2 had no history of sinus disease despite being referred under the diagnostic construct of limited GPA. Patient 3 developed a novel inflammatory syndrome suspected to represent GPA shortly after having been treated with rituximab (an effective GPA therapy); however, he lacked sinus or pulmonary symptoms despite having pulmonary findings on CT imaging. Additionally, all three of our patients yielded negative or incongruent ANCA results, along with refractory symptoms that required long-term glucocorticoid therapy. These cases highlight the need for rheumatologists to consider VEXAS syndrome as a potential aetiology in refractory and/or atypical cases of suspected AAV arising in older male patients, and rarely in females with somatic mosaicism or skewed X-inactivation [5].

**Table 1.** Characteristics of patients with VEXAS syndrome and AAV phenotype

Characteristics	Patient								
	1	2	3	4	5	6	Our patient 1	Our patient 2	Our patient 3
Age (years)	72	70	71	68	55	76	72	88	77
AAV phenotype	MPA	GPA	GPA	GPA	GPA	GPA	EGPA	GPA	GPA
Antibody profile	MPO^+^	PR3^+^	C-ANCA^+^/PR3^+^	ANCA^−^	ANCA^−^	PR3^+^	ANCA^−^	ANA 1:80 speckled, ANCA^−^	ANA 1:320, C-ANCA^+^
Renal biopsy	Necrotizing crescentic glomerulonephritis	Peritubular capillaritis	NA	Pauci-immune glomerulonephritis	NA	NA	NA	NA	Glomerulosclerosis, diffuse moderate tubular injury, moderate tubulointerstitial scarring
*UBA1* mutation	p.Met41Val	p.Met41Thr	+	p.Met41Thr	p.Met41Val	p.Met41Thr	p.Met41Thr	p.Met4Leu	p.Met41Thr
Macrocytosis	Yes	Yes	Yes	Yes	Yes	Yes	Yes	Yes	Yes
Recurrent fever	Yes	Yes	No	Yes	Yes	Yes	Yes	No	No
Lung compromise	Pulmonary infiltrates (GGOs)	No	No	Multiple subpleural pulmonary nodules and GGOs	Pulmonary infiltrates (GGOs), biopsy showed lymphohistioplasmocellular vasculitis of larger arterioles	Pulmonary nodules and GGOs	Pulmonary infiltrates (GGOs)	No	Pulmonary nodules
Skin findings	No	No	Skin plaque with multinucleated oval cell infiltrate	No	Leukocytoclastic vasculitis	Leukocytoclastic vasculitis	Medium-vessel cutaneous vasculitis with intravascular eosinophils	No	Pemphigus
Other key features	Bone marrow with vacuoles	Ocular inflammation, inflammatory arthritis, bone marrow with vacuoles	No	Nasal septum perforation, chronic sinusitis, bone marrow with vacuoles	Nasal ulcers	Bilateral scleritis	Nasal polyps, eosinophilia, weight loss	Polyarthritis, ocular inflammation, chondritis	Ocular inflammation, inflammatory arthritis
Inflammatory markers	CRP 250 mg/dl, ESR 137 mm/h	NA	NA	CRP 300 mg/dl	CRP 153 mg/dl	CRP 13.3 mg/dl	CRP 24 mg/dl	ESR 125 mm/h, CRP 14.8 mg/dl	ESR 100 mm/h, CRP 7 mg/dl
Treatment	CYC, MMF, RTX, GCs	RTX, MMF, IFX, TCZ, TOFA, DEC, GCs	MMF, MTX, AZA, IFX, RTX, TCZ GCs	GCs, CYC, RTX, TCZ	GCs, CYC, Anakinra, RTX	GCs, TCZ, MTX, IFX	GCs, AZA, Mepolizumab, MTX. TCZ	GCs, MTX	GCs, RTX
Reference	[7]	[8]	[9]	[10]	[11]	[12]			

DEC: decitabine, GCs: glucocorticoids; IFX: infliximab; RTX: rituximab; TCZ: tocilizumab; TOFA: tofacitinib.

A recent review by Bruno *et al.* characterized the inflammatory manifestations of VEXAS syndrome, revealing that, in addition to constitutional symptoms, the most common features shared between VEXAS and autoimmune conditions, ranked by frequency, include skin findings (neutrophilic dermatosis, leukocytoclastic vasculitis and septal panniculitis), lung involvement (dyspnoea, cough and multiple radiological findings) and ocular manifestations (uveitis, scleritis, episcleritis and retinal vasculitis) [3]. The AAV cases we have compiled align with these patterns, with a majority exhibiting pulmonary infiltrates [67% (6/9)], skin involvement [55% (5/9)] and ocular manifestations [44% (4/9)]. Although renal compromise is typically observed in AAV, only 44% (4/9) of patients in our study had renal involvement, and among them, only 2 displayed kidney lesions resembling the typical AVV pattern (pauci-immune focal necrotizing glomerulonephritis) [13], while the other 2 exhibited non-specific tubular compromise. Therefore, clinicians should consider the possibility of VEXAS in cases of atypical kidney damage, even when clinical features may initially resemble AAV—bearing in mind that renal histology is among the most specific markers of AAV [14].

The immunological basis for an AAV phenotype within the spectrum of VEXAS syndrome remains poorly understood. However, it has been proposed that the dysregulated activation of neutrophils and the generation of neutrophil extracellular traps (NETs) may be a shared feature in these two conditions. A pathogenic role for ANCA in inducing neutrophil activation in AAV has been demonstrated [13]. In VEXAS, disruption of the ubiquitylation pathway in myeloid cells causes unfolded proteins to accumulate in the cytosol, which leads to sustained endoplasmic reticulum stress and an unfolded protein response (UPR) via the PERK, IRE1α and ATF6 sensors [15] This UPR is accompanied by a dysregulated pro-inflammatory response mediated by the TNF, IL-1, IL-6, IL-8 and IFN-γ pathways, resulting in aberrant neutrophil activation and endothelitis [16–18]. Furthermore, IRE1α has been identified as a key regulator of NET formation, and its inhibition could potentially mitigate an immune complex–mediated NETosis [19]. The interplay between neutrophil activation and the UPR may represent a relevant therapeutic target in VEXAS with an AAV phenotype.

As of yet, there are no standardized guidelines for the identification or treatment of different VEXAS phenotypes, despite the fact that this syndrome can manifest with a broad range of clinical features. Additional studies aimed at identifying atypical and/or refractory cases of what was initially thought to represent AAV, but later proven to be VEXAS syndrome, will increase our understanding of the clinical heterogeneity encompassed by this clonal process. Such investigations may contribute not only to our ability to identify VEXAS syndrome at an earlier stage in the disease course, but also to identify improved treatment strategies for this challenging clinical entity.

## Data Availability

The data underlying this article will be shared upon reasonable request to the corresponding author.
